# Dataset on blood parameters including leukocyte profiles of eastern European vespertilionid and rhinolophid bats

**DOI:** 10.1038/s41597-025-06274-0

**Published:** 2025-12-10

**Authors:** Ihor Tovstukha, Valeriia Bohodist, Denys Muzyka, Viktoriia Radchuk, Kseniia Kravchenko, Anton Vlaschenko

**Affiliations:** 1 Ukrainian Bat Rehabilitation Center, NGO “Ukrainian Independent Ecology Institute”, Heorgia Tarasenko st., 40, 61001 Kharkiv, Ukraine; 2 Kharkiv International Medical University, Molochna St, 38, 61001 Kharkiv, Ukraine; 3 Educational and Research Bat Biology Laboratory, H.S. Skovoroda Kharkiv National Pedagogical University, Institute of Natural Sciences, Valentynivska St. 2, Kharkiv, 61168 Ukraine; 4 National Scientific Center “Institute of Experimental and Clinical Veterinary Medicine”, H. Skovorody St., 83, Kharkiv, 61023 Ukraine; 5 Leibniz Institute for Zoo and Wildlife Research, Alfred-Kowalke-Straße 17, 10315 Berlin, Germany; 6 Faculty of Science, Technology and Medicine, University of Luxembourg Belval Campus 2, de l’Université, L-4365, Esch-sur-Alzette, Luxembourg

**Keywords:** Immunology, Zoology

## Abstract

Understanding the immunological profiles of wild bats is crucial for monitoring population health, disease dynamics, and stress responses in changing environments. Here, we present a dataset of 747 blood smear samples collected from 19 European bat species between 2017 and 2023 in Ukraine. For a substantial portion of individuals, red and white blood cell counts were assessed, and for most, differential leukocyte profiles were recorded, including segmented and band neutrophils, lymphocytes, monocytes, eosinophils, and basophils. The dataset is accompanied by rich metadata, which, among other aspects, includes species, sex, age class, life-cycle stage (e.g., hibernation, migration, breeding), capture location, habitat type and others. Most individuals were sampled during the hibernation period in urban environments. The dataset offers a valuable baseline for comparative hematology, eco-immunology, and bat conservation research. By sharing detailed leukocyte profiles and standardized metadata, this dataset facilitates cross-species comparisons, contributes to wildlife health surveillance, and supports future studies on the impacts of environmental stressors and zoonotic risks in European bats.

## Background & Summary

Bats are a remarkably diverse group of volant mammals, with over 1,400 described species worldwide^1^. They are globally distributed, and inhabit most of the Earth’s biomes, except for the Arctic and Antarctic ice deserts. Bats demonstrate a wide range of feeding strategies, including insectivory, frugivory, nectarivory, carnivory, and sanguivory, reflecting their adaptation to diverse ecological niches^2^. This dietary diversity not only distinguishes them from other mammals but also leads to significant variation in physiological traits within the order Chiroptera, contributing to differences in metabolism^3^, immune function^4^, and reproductive strategies^5^.

Bats are reservoirs for a wide range of pathogens, especially viruses. The ecological diversity, social behaviors, and mobility of bats promote contact with viruses such as lyssaviruses, filoviruses, coronaviruses and others. Remarkably, bats often harbor these pathogens without showing clinical signs, positioning them as key players in pathogen maintenance and potential spillover^6^. This capacity for viral tolerance is linked to unique features of the bat immune system. Elevated baseline interferon expression, rapid antiviral gene activation, and a controlled inflammatory response allow bats to suppress viral replication^7^ while avoiding tissue damage. These traits may have evolved alongside the high metabolic demands of flight, resulting in an immune system optimized for resilience and regulation.

While there are currently no known mass mortality events in bats caused by viral infections, their immune system appears much less resilient to bacterial and, in particular, fungal infections, which are often the cause of severe diseases^8^. One striking example is white-nose syndrome, which has killed millions of bats during the last decades across North America. This highlights striking features of the bat immune defense system that differentiate them from other mammals and emphasizes the need for a better understanding of how the bat immune system functions^9,10^.

Understanding the cellular components of bat immunity - particularly through blood parameters such as leukocyte profiles - can offer insights into how bats respond to diverse pathogens under natural conditions. Leukocyte differentials offer a valuable window into the immune health of individuals and populations, e.g.:^10,11^. However, standardized, large-scale haematological data across species and regions^12^ remain scarce, especially in temperate zones. In tropical regions, such blood parameters have been employed to explore how environmental disturbances, such as deforestation or habitat fragmentation, influence bat health^10^.

In the frame of bat rehabilitation activity^13–16^, we have rescued hundreds of weakened bats that show no signs of trauma or external injuries. One of the primary methods to determine whether these bats are weakened due to exhaustion or underlying pathogens and parasites is through leukocyte profile analysis. However, there is a lack of reference data for leukocyte profiles in European bats. This gap not only limits our ability to assess the health status of rescued bats but also challenges researchers studying bat immunology and physiology across Europe. Without adequate baseline data on leukocyte profiles, it is difficult to fully understand the impact of pathogens, parasites, and environmental stressors on European bat populations. Addressing this gap became our primary motivation for collecting as many leukocyte profiles of European bats as possible from the territory of Ukraine. Here, we present a dataset of over 700 individual bat leukocyte profiles^17^, which will significantly contribute to advancing knowledge in this area. We performed a detailed analysis of 747 blood smears from European bats (Table 1)^17^, focusing on their leukocyte profiles and total red (RBC) and white (WBC) blood cell count (Tables 2, 3). These data were collected from various regions across Ukraine and represent nineteen bat species (seven of them with sample-size more than 25 individuals)^17^. This dataset provides a reference for future studies on bat health populations in Europe.

**Table 1:** Summary of sample size (n = number of individuals) by bat species and blood parameters (RBC - red blood cells, WBC - white blood cells).

Bat species name	RBC (n)	WBC (n)	Leukocyte profiles (n)
Lesser horseshoe bat (*Rhinolophus hipposideros*) n = 10	0	0	10
Barbastelle bat (*Barbastella barbastellus*) n = 14	1	1	14
Common serotine bat (*Eptesicus serotinus*) n = 105	47	65	104
Alcathoe bat (*Myotis alcathoe*) n = 7	0	0	7
Bechstein’s bat (*Myotis bechsteinii*) n = 6	0	0	6
Lesser mouse-eared bat (*Myotis blythii*) n = 5	0	0	5
Pond bat (*Myotis dasycneme*) n = 4	0	1	4
Daubenton’s bat (*Myotis daubentonii*) n = 80	11	23	80
Greater mouse-eared bat (*Myotis myotis*) n = 15	0	0	15
Natterer’s bat (*Myotis nattereri*) n = 6	0	0	6
Greater noctule (*Nyctalus lasiopterus*) n = 2	2	2	2
Leisler’s noctule (*Nyctalus leisleri*) n = 4	1	1	4
Common noctule (*Nyctalus noctula*) n = 298	60	144	296
Kuhl’s pipistrelle (*Pipistrellus kuhlii lepidus*) n = 50	6	6	50
Nathusius’ pipistrelle (*Pipistrellus nathusii*) n = 58	27	40	58
Soprano pipistrelle (*Pipistrellus pygmaeus*) n = 13	0	0	13
Brown long-eared bat (*Plecotus auritus*) n = 38	11	17	36
Gray long-eared bat (*Plecotus austriacus*) n = 4	0	0	4
Parti-coloured bat (*Vespertilio murinus*) n = 28	19	25	28

**Table 2:** Summary of mean and median values of RBC (red blood cells, 10^12^ cells/L) and WBC (white blood cells, 10^9^ cells/L) for bat species with a sample size of more than three individuals (n = number of individuals).

Bat species name	RBC median/mean ± SD, min, max (n)	WBC median/mean ± SD, min, max (n)
*E. serotinus*	8.60/8.125 ± 2.73, 3.6, 14.8 (47)	4.80/5.35 ± 2.80, 0.9, 12.0 (65)
*M. daubentonii*	10.00/10.35 ± 1.90, 7.5, 13.3 (11)	4.10/4.80 ± 3.03, 0.8, 13.5 (23)
*N. noctula*	10.40/10.54 ± 3.02, 3.3, 23.0 (60)	4.25/5.18 ± 3.64, 0.0, 20.2 (144)
*P. k. lepidus*	9.15/9.13 ± 0.99, 7.9, 10.5 (6)	5.45/6.02 ± 3.41, 2.7, 12.5 (6)
*P. nathusii*	7.90/8.01 ± 1.99, 4.8, 13.2 (27)	4.80/5.42 ± 2.42, 2.1, 13.4 (40)
*Pl. auritus*	13.00/12.43 ± 3.18, 6.3, 16.0 (11)	2.50/2.97 ± 2.33, 0.2, 10.4 (17)
*V. murinus*	11.00/11.01 ± 2.32, 7.0, 14.8 (19)	6.80/6.54 ± 2.22, 2.7, 10.5 (25)

**Table 3:** Summary of mean and median values (%) of Leukocyte profiles (SN - segmented neutrophils; BN - band neutrophils; L - lymphocytes; M - monocytes; EO - eosinophils; BA - basophils) for twelve bat species in the dataset (n = number of individuals).

Bat species name	SN% median/mean ± SD, min, max	BN% median/mean ± SD, min, max	L% median/mean ± SD, min, max	M% median/mean ± SD, min, max	EO% median/mean ± SD, min, max	BA% median/mean ± SD, min, max
*Rh. hipposideros* n = 10	19.0/18.1 ± 10.3, 4.0, 36.0	0.0/0.2 ± 0.4, 0.0, 1.0	80.0/79.5 ± 11.1, 61.0, 93.0	1.5/2.0 ± 1.2, 1.0, 4.0	0.0/0.1 ± 0.3, 0.0, 1.0	0.0/0.2 ± 0.4, 0.0, 1.0
*B. barbastellus* n = 14	35.0/41.6 ± 15.9, 22.0, 67.0	0.5/0.6 ± 0.7, 0.0, 2.0	62.5/55.5 ± 16.6, 28.0, 75.0	2.0/1.6 ± 1.3, 0.0, 4.0	0.0/0.4 ± 0.5, 0.0, 1.0	0.0/0.2 ± 0.4, 0.0, 1.0
*E. serotinus* n = 104	40.5/40.7 ± 17.4, 6.0, 80.0	1.0/1.4 ± 1.2, 0.0, 5.0	53.0/53.1 ± 17.8, 18.0, 93.0	3.0/4.2 ± 3.4, 0.0, 15.0	0.0/0.5 ± 0.7, 0.0, 4.0	0.0/0.1 ± 0.3, 0.0, 1.0
*M. alcathoe* n = 7	10.0/11.4 ± 6.5, 6.0, 24.0	0	89.0/87.4 ± 6.8, 75.0, 94.0	1.0/1.0 ± 0.8, 0.0, 2.0	0.0/0.4 ± 0.5, 0.0, 1.0	0
*M. bechsteinii* n = 6	19.0/21.3 ± 9.9, 8.0, 34.0	0.0/0.3 ± 0.5, 0.0, 1.0	78.5/76.0 ± 10.8, 63.0, 91.0	1.5/1.3 ± 0.8, 0.0, 2.0	0.0/0.3 ± 0.5, 0.0, 1.0	1.0/0.7 ± 0.5, 0.0, 1.0
*M. blythii* n = 5	38.0/43.6 ± 20.8, 24.0, 67.0	0.0/0.4 ± 0.5, 0.0, 1.0	60.0/54.0 ± 22.3, 29.0, 75.0	1.0/1.4 ± 1.1, 0.0, 3.0	0.0/0.4 ± 0.5, 0.0, 1.0	0.0/0.2 ± 0.4, 0.0, 1.0
*M. dasycneme* n = 4	31.0/31.2 ± 9.9, 20.0, 43.0	1.0/1.0 ± 0.8, 0.0, 2.0	64.5/64.5 ± 9.6, 53.0, 76.0	1.5/1.8 ± 1.0, 1.0, 3.0	1.0/1.5 ± 1.0, 1.0, 3.0	0
*M. daubentonii* n = 80	34.5/36.8 ± 14.8, 10.0, 70.0	1.0/0.9 ± 0.8, 0.0, 4.0	62.0/58.7 ± 15.6, 23.0, 85.0	2.0/2.9 ± 2.4, 0.0, 11.0	0.0/0.5 ± 0.7, 0.0, 3.0	0.0/0.2 ± 0.4, 0.0, 1.0
*M. myotis* n = 15	36.0/38.6 ± 19.7, 12.0, 70.0	1.0/0.7 ± 0.7, 0.0, 2.0	58.0/58.2 ± 20.1, 26.0, 86.0	1.0/1.9 ± 1.4, 0.0, 5.0	0.0/0.5 ± 0.5, 0.0, 1.0	0.0/0.2 ± 0.4, 0.0, 1.0
*M. nattereri* n = 6	69.5/62.3 ± 31.4, 14.0, 94.0	1.0/0.8 ± 0.8, 0.0, 2.0	28.0/35.0 ± 31.3, 5.0, 85.0	1.0/1.0 ± 0.9, 0.0, 2.0	0.5/0.5 ± 0.5, 0.0, 1.0	0.0/0.2 ± 0.4, 0.0, 1.0
*N. lasiopterus* n = 2	37.0/37.0 ± 5.7, 33.0, 41.0	2.5/2.5 ± 0.7, 2.0, 3.0	51.5/51.5 ± 2.1, 50.0, 53.0	8.5/8.5 ± 3.5, 6.0, 11.0	0.0/0.0 ± 0.0, 0.0, 0.0	0.5/0.5 ± 0.7, 0.0, 1.0
*N. leisleri* n = 4	48.0/50.8 ± 9.7, 43.0, 64.0	0.0/1.0 ± 2.0, 0.0, 4.0	46.0/45.2 ± 9.4, 33.0, 56.0	2.0/2.8 ± 2.2, 1.0, 6.0	0.0/0.2 ± 0.5, 0.0, 1.0	0

**Table 4:** Summary of mean and median values (%) of Leukocyte profiles (SN - segmented neutrophils; BN - band neutrophils; L - lymphocytes; M - monocytes; EO - eosinophils; BA - basophils) for seven bat species in the dataset (n = number of individuals).

Bat species name	SN% median/mean ± SD, min, max	BN% median/mean ± SD, min, max	L% median/mean ± SD, min, max	M% median/mean ± SD, min, max	EO% median/mean ± SD, min, max	BA% median/mean ± SD, min, max
*N. noctula* n = 296	40.5/42.3 ± 17.8, 9.0, 88.0	1.0/1.2 ± 1.0, 0.0, 8.0	54.0/52.9 ± 18.5, 6.0, 90.0	3.0/3.1 ± 2.3, 0.0, 12.0	0.0/0.5 ± 0.7, 0.0, 6.0	0.0/0.3 ± 0.5, 0.0, 2.0
*P. k. lepidus* n = 50	63.5/62.8 ± 14.4, 29.0, 89.0	1.0/0.8 ± 0.6, 0.0, 2.0	30.5/33.0 ± 14.7, 7.0, 66.0	2.0/2.6 ± 1.8, 1.0, 7.0	0.0/0.4 ± 0.6, 0.0, 2.0	0.0/0.4 ± 0.5, 0.0, 2.0
*P. nathusii* n = 58	33.5/35.2 ± 11.9, 16.0, 64.0	1.0/1.4 ± 1.2, 0.0, 5.0	61.5/59.3 ± 12.6, 27.0, 82.0	3.0/3.3 ± 2.3, 0.0, 10.0	0.0/0.3 ± 0.6, 0.0, 2.0	0.0/0.4 ± 0.6, 0.0, 2.0
*P. pygmaeus* n = 13	51.0/53.5 ± 12.4, 30.0, 79.0	1.0/1.1 ± 0.8, 0.0, 2.0	46.0/43.2 ± 12.6, 18.0, 66.0	2.0/1.5 ± 1.2, 0.0, 4.0	1.0/0.5 ± 0.5, 0.0, 1.0	0.0/0.1 ± 0.3, 0.0, 1.0
*Pl. auritus* n = 36	33.5/32.6 ± 14.2, 7.0, 68.0	1.0/1.1 ± 1.1, 0.0, 3.0	62.0/62.3 ± 15.4, 27.0, 91.0	2.0/3.6 ± 2.9, 1.0, 11.0	0.0/0.2 ± 0.5, 0.0, 2.0	0.0/0.2 ± 0.4, 0.0, 1.0
*Pl. austriacus* n = 4	42.0/37.8 ± 13.7, 18.0, 49.0	0.5/0.5 ± 0.6, 0.0, 1.0	54.5/60.2 ± 14.7, 50.0, 82.0	1.0/1.0 ± 1.2, 0.0, 2.0	1.0/0.8 ± 0.5, 0.0, 1.0	0
*V. murinus* n = 28	27.0/30.2 ± 13.8, 9.0, 63.0	2.0/1.9 ± 1.4, 0.0, 5.0	66.0/62.8 ± 15.3, 34.0, 82.0	4.0/4.1 ± 2.0, 1.0, 9.0	0.0/0.3 ± 0.6, 0.0, 2.0	0.0/0.3 ± 0.5, 0.0, 2.0

## Methods

### Ethical statements

All applicable international, national, and institutional guidelines for the care and use of animals were followed^15,18^. The study was approved by the following protocols of ethic committees in different years: 2017 the Ethics Committee of V.N. Karazin Kharkiv University (Decision #03/2017), in 2022–2024 by the Ministry of Environmental Protection and Natural Resources for the project Р781 (DTRA, HDTRA1-21-1-0043) and in 2022 by the Bio-ethics Committee of National Scientific Center “Institute of Experimental and Clinical Veterinary Medicine” (protocol number: 1-22b 15.02.2022). A specific protocol for monitoring blood clotting and checking animal health has been developed (see: section “Blood collection and sample preparation” below). No animals died or were found to be too weak to fly independently following the sampling procedure.

### Capture and handling

Wild bats were captured using mist nets^18^, and two standardized workflows were followed: immediate sampling within 1–3 hours of capture, or short holding in cloth bags kept in a cool, draft-free room protected from direct sunlight with sampling at 7–18 hours post-capture. At the rehabilitation centre facilities, bats were watered, underwent basic clinical checks, and were weighed^19^. Afterwards, individuals were placed into artificial hibernation (refrigerated units) following rehabilitation protocols^20^. Bats that remained in continuous torpor between 10 days and up to 2 months were considered for sampling (individuals that were fed during hibernation were excluded). For blood collection, bats were gently aroused and sampled 12–24 hours after arousal.

Age class (subadult and adult) was assigned using standard criteria^15,18,19^ (e.g., epiphyseal fusion of the fingers, tooth wear, reproductive status). Month-level age (CertainAge parameter)^17^ was recorded in two situations: (i) subadult (this-year-born individuals) - estimated by simple calculation from the regional birth window (e.g., pups assumed born in June: an individual collected in August was recorded as 2 months old); (ii) known-age ringed bats - banding/recapture dates provided a precise age of sampling individual. If neither criterion was met, CertainAge was set to NA^17^.

### Blood collection and sample preparation

Venous blood samples were collected from live, free-ranging bats following ethical guidelines and established field protocols^21–24^. Individuals in poor condition, including underweight or recently volant juvenile bats, were excluded from sampling. Blood was obtained via venipuncture of the propatagial or femorocaudal vein using sterile 27 G needles. The puncture site was disinfected with 70% ethanol prior to sampling. Up to 17 μl of blood was collected per individual using a sterile micropipette with disposable 1–10 μl tips and was divided into three aliquots for further analyses.

Blood sampling procedures avoided excessive blood loss for bats: for each bat, the sampled blood volume was capped at ≤0.5% of body mass, i.e., half of the commonly recommended ≤1% limit for small mammals^25^. We did not sample visibly ill, injured, dehydrated, emaciated, or very young individuals. Venipuncture was performed at sites that allow effective compression hemostasis using fine-gauge needles while avoiding repeated punctures. Immediately after sampling, the puncture site was compressed with two cotton swabs moistened with 70% ethanol - one directly over and one opposite the vein - to apply moderate, even pressure for ~5 min (extended as needed until hemostasis).

If bleeding persisted, the swabs were replaced with new ones soaked in 3% hydrogen peroxide, and compression was continued, occasionally extending up to 15 minutes. In cases of active or prolonged bleeding, 5% aminocaproic acid solution was applied locally (to moisten the swab) and administered orally (5 ml/kg body weight). In more severe cases, subcutaneous injections of the same solution were used (up to 2.5 ml/kg). When necessary, Celox (chitosan-based hemostatic powder) was applied locally and compressed with a dry swab. These measures were effective in achieving hemostasis in all recorded cases following venipuncture. After blood collection, all bats were offered water ad libitum. In cases where bleeding did not stop immediately, individuals were additionally provided with a glucose solution, and in some instances, they were fed mealworm larvae (*Tenebrio molitor*). Before release, each bat was carefully re-examined to ensure that bleeding had not resumed and that the animal was capable of flight.

### Erythrocyte and leukocyte counts

To estimate total red blood cell (RBC) counts, 10 μl of blood was diluted 1:200 in 3% sodium chloride solution (Sigma-Aldrich, St. Louis, MO, USA). The mixture was loaded into a Goryaev chamber and erythrocytes were counted in five large squares under 150 × magnification. The resulting cell count was multiplied by 10,000 (as determined by the hemocytometer’s characteristics) to obtain the final value, expressed as 10¹² cells per liter.

For total white blood cell (WBC) counts, 5 μl of blood was diluted 1:20 in 3% acetic acid solution (Sigma-Aldrich, St. Louis, MO, USA) and examined in a Goryaev chamber under similar magnification. Leukocytes were counted in 100 large squares, and the resulting number was multiplied by 50 (as determined by the hemocytometer’s characteristics) to obtain the final value, expressed as 10⁹ cells per liter.

### Blood smear preparation and differential leukocyte count

The remaining 7 μl of blood was used to prepare smears on microscope slides. After air-drying, smears were fixed with methanol and stained using Romanowsky-Giemsa stain. Differential leukocyte counts (WBCd) were performed under light microscopy (1350x magnification using oil immersion) by identifying 100 leukocytes (50 in a few cases) and classifying them as segmented or band neutrophils, lymphocytes, monocytes, eosinophils, or basophils based on standard morphological and tinctorial features. The relative proportions were calculated and, where possible, absolute values were derived by multiplying the proportion by the total WBC count. All smears were analyzed blind by a single observer (Tovstukha I) to ensure consistency.

## Data Records

The dataset described here is openly available at Zenodo (10.5281/zenodo.15648835)^17^ and contains individual-level measurements of red and white blood cell counts and leukocyte differentials, accompanied by a data dictionary and README describing variables and units. The dataset is provided in Excel (.xlsx) format.

**Fig. 1:**
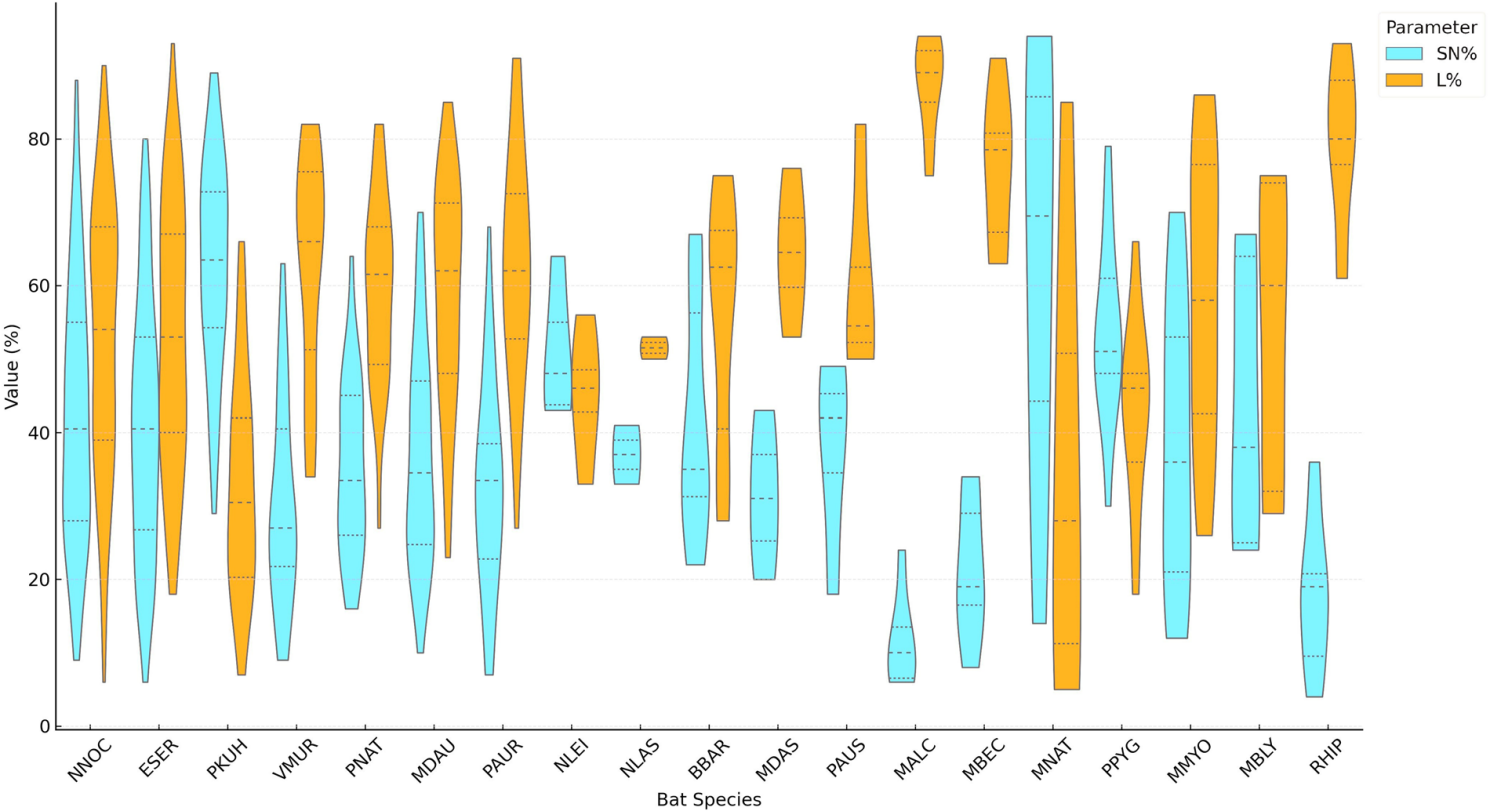
Violin plot of Leukocyte profiles of lymphocytes (L) and segmented neutrophils (SN) in examined bat blood smears, center dashed line - median, the upper and lower lines are the limits of the interquartile (25% and 75%) range (NNOC, ESER, … abbreviations of species names: the first letter of the genus name and the first three letters of the species name).

**Fig. 2:**
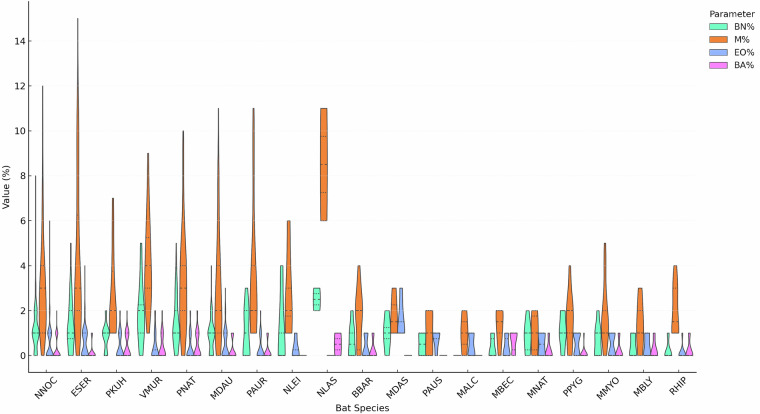
Violin plot of Leukocyte profiles of band neutrophils (BN), monocytes (M), eosinophils (EO), and basophils (BA) in examined bat blood smears, center dashed line - median, the upper and lower lines are the limits of the interquartile (25% and 75%) range (NNOC, ESER, … abbreviations of species names: the first letter of the genus name and the first three letters of the species name).

Each row represents a single individual and contains information on species, sex, age, sampling location, and detailed leukocyte profiles^17^. Blood parameters include red and white blood cell counts, along with the relative percentages of neutrophils (segmented and band), lymphocytes (Fig. 1), monocytes, eosinophils, and basophils (Fig. 2). Additional metadata^17^ include the bat’s life stage at sampling (e.g., hibernation, breeding) (Table 8, Supplementary Table S3), sex-age groups (Tables 5, 6, Supplementary Table S1), capture status (wild or rehabilitated), geographic coordinates, habitat type (Table 7, Supplementary Table S2), and for a subset of individuals from the Chernobyl Exclusion Zone^18^, body concentrations of **⁹⁰**Sr and **¹³⁷**Cs are provided. Individuals with radioactive isotopes are not shown in the main tables here to preserve readability.

**Table 5:** Summary of mean and median values (n = number of individuals) of RBC (red blood cells, 10^12^ cells/L) and WBC (white blood cells, 10^9^ cells/L) for different sex-age groups of four bat species, with a sample size of more than 25 individuals (in total).

Bat species name	Sex and age group	RBC median/mean ± SD, min, max (n)	WBC median/mean ± SD, min, max (n)
*N. noctula*	F_total	9.7/10.2 ± 2.5, 3.3, 14.3 (22)	4.2/5.2 ± 3.7, 1.2, 20.2 (63)
	F_ad	9.5/9.9 ± 2.5, 3.3, 13.6 (13)	3.7/4.0 ± 1.8, 1.4, 9.5 (37)
	F_sad	9.8/10.6 ± 2.6, 7.0, 14.3 (9)	5.3/6.9 ± 4.8, 1.2, 20.2 (26)
	M_total	11.0/10.7 ± 3.3, 4.5, 23.0 (38)	4.3/5.1 ± 3.7, 0.0, 19.8 (81)
	M_ad	11.0/10.8 ± 2.6, 4.5, 16.0 (21)	3.7/3.9 ± 2.1, 1.2, 10.4 (32)
	M_sad	10.0/10.7 ± 4.1, 6.0, 23.0 (17)	4.8/6.0 ± 4.2, 0.0, 19.8 (49)
*E. serotinus*	F_total	7.9/8.5 ± 3.0, 4.8, 13.2 (8)	4.9/5.4 ± 2.6, 1.8, 11.0 (22)
	F_ad	10.0/10.0 ± 2.9, 6.8, 13.2 (4)	5.0/5.6 ± 2.7, 1.8, 11.0 (17)
	F_sad	6.3/6.9 ± 2.5, 4.8, 10.3 (4)	3.9/4.7 ± 2.7, 2.2, 9.2 (5)
	M_total	8.8/8.1 ± 2.7, 3.6, 14.8 (39)	4.6/5.3 ± 2.9, 0.9, 12.0 (43)
	M_ad	8.8/8.1 ± 2.7, 3.6, 14.8 (36)	4.6/5.2 ± 2.9, 0.9, 12.0 (39)
	M_sad	6.3/7.3 ± 2.6, 5.3, 10.3 (3)	6.0/6.2 ± 3.1, 3.0, 9.8 (4)
*M. daubentonii*	F_total	9.5/9.0 ± 1.3, 7.5, 10.3 (5)	3.7/5.0 ± 3.3, 1.9, 11.6 (8)
	F_ad	N/A	7.5/7.5 ± 5.7, 3.5, 11.6 (2)
	F_sad	9.5/9.0 ± 1.3, 7.5, 10.3 (5)	3.7/4.1 ± 2.2, 1.9, 7.9 (6)
	M_total	11.7/11.5 ± 1.6, 9.3, 13.3 (6)	4.2/4.7 ± 3.0, 0.8, 13.5 (15)
	M_ad	12.4/12.4 ± 1.3, 11.5, 13.3 (2)	3.7/3.5 ± 2.1, 0.8, 5.8 (5)
	M_sad	10.9/11.0 ± 1.7, 9.3, 13.0 (4)	4.2/5.3 ± 3.5, 1.1, 13.5 (9)
*P. nathusii*	F_total	8.2/8.3 ± 2.4, 4.8, 13.2 (14)	4.7/5.0 ± 2.0, 2.4, 9.3 (21)
	F_ad	8.6/9.3 ± 3.6, 6.2, 13.2 (3)	4.9/6.0 ± 2.2, 4.7, 9.3 (4)
	F_sad	7.8/8.0 ± 2.2, 4.8, 13.0 (11)	4.2/4.8 ± 2.0, 2.4, 8.8 (17)
	M_total	7.9/7.7 ± 1.4, 6.0, 10.8 (13)	5.2/5.9 ± 2.8, 2.1, 13.4 (19)
	M_ad	8.4/8.2 ± 1.8, 6.1, 10.8 (5)	4.5/5.8 ± 3.8, 3.0, 13.4 (7)
	M_sad	7.4/7.5 ± 1.3, 6.0, 9.3 (6)	6.2/6.0 ± 2.4, 2.1, 10.7 (10)

**Table 6:** Summary of mean and median values (n = number of individuals) of RBC (red blood cells, 10^12^ cells/L) and WBC (white blood cells, 10^9^ cells/L) for different sex-age groups of three bat species, with a sample size of more than 25 individuals (in total).

Bat species name	Sex and age group	RBC median/mean ± SD, min, max (n)	WBC median/mean ± SD, min, max (n)
*P. k. lepidus*	F_total	8.7/8.7 (1)	4.0/4.0 (1)
	F_ad	8.7/8.7 (1)	4.0/4.0 (1)
	F_sad	N/A	N/A
	M_total	9.6/9.2 ± 1.1, 7.9, 10.5 (5)	5.9/6.4 ± 3.6, 2.7, 12.5 (5)
	M_ad	9.6/9.2 ± 1.1, 7.9, 10.5 (5)	5.9/6.4 ± 3.6, 2.7, 12.5 (5)
	M_sad	N/A	N/A
*Pl. auritus*	F_total	13.7/13.9 ± 1.6, 12.3, 16.0 (4)	2.7/3.7 ± 3.3, 0.2, 10.4 (7)
	F_ad	14.5/14.5 ± 2.1, 13.0, 16.0 (2)	2.4/2.5 ± 1.7, 0.2, 4.6 (5)
	F_sad	12.3/12.3 ± nan, 12.3, 12.3 (1)	10.4/10.4 (1)
	M_total	13.0/11.6 ± 3.6, 6.3, 14.5 (7)	2.5/2.5 ± 1.3, 0.5, 5.3 (10)
	M_ad	14.0/13.7 ± 0.8, 12.6, 14.5 (5)	2.5/2.4 ± 1.5, 0.5, 5.3 (7)
	M_sad	6.4/6.4, 6.4, 6.4 (1)	2.1/2.1 ± 0.6, 1.7, 2.5 (2)
*V. murinus*	F_total	11.3/11.2 ± 2.5, 7.0, 14.8 (11)	6.8/6.3 ± 2.0, 2.7, 9.5 (14)
	F_ad	10.8/10.8 ± 3.2, 8.5, 13.0 (2)	6.8/6.8 ± 3.8, 4.1, 9.5 (2)
	F_sad	11.0/10.5 ± 2.4, 7.0, 13.9 (7)	6.6/6.1 ± 2.0, 2.7, 8.7 (10)
	M_total	10.3/10.8 ± 2.1, 7.7, 13.8 (8)	6.8/6.8 ± 2.5, 2.8, 10.5 (11)
	M_ad	8.2/8.2 ± 0.8, 7.7, 8.8 (2)	6.8/6.6 ± 3.3, 3.2, 9.7 (3)
	M_sad	11.0/11.4 ± 1.7, 10.0, 13.8 (4)	7.9/7.4 ± 2.6, 2.8, 10.5 (6)

**Table 7:** Summary of mean and median values of RBC (red blood cells, 10^12^ cells/L) and WBC (white blood cells, 10^9^ cells/L) for different Habitats of seven bat species, with a sample size of more than 25 individuals (in total) (n = number of individuals).

Bat species name	Habitat type	RBC median/mean ± SD, min, max (n)	WBC median/mean ± SD, min, max (n)
*N. noctula*	Natural	4.5/5.7 ± 3.17, 3.3, 9.3 (3)	2.95/3.28 ± 1.26, 1.4, 5.8 (26)
	Rural	10.15/11.29 ± 3.43, 7.0, 23.0 (22)	8.1/8.97 ± 4.68, 3.0, 20.2 (23)
	Urban	10.75/10.15 ± 2.18, 6.0, 13.3 (28)	3.75/4.64 ± 3.13, 0.0, 13.9 (88)
*E. serotinus*	Natural	6.0/6.79 ± 2.43, 4.5, 11.3 (9)	3.1/3.52 ± 1.78, 0.9, 7.6 (16)
	Rural	7.6/7.58 ± 2.81, 3.6, 13.2 (23)	7.2/7.32 ± 2.68, 3.4, 12.0 (24)
	Urban	9.3/9.76 ± 2.04, 6.0, 14.8 (15)	4.5/4.63 ± 2.33, 2.0, 11.0 (25)
*M. daubentonii*	Natural	10.15/10.45 ± 1.97, 7.5, 13.3 (10)	3.7/4.32 ± 2.44, 0.8, 11.6 (21)
	Rural	9.3/9.3, 9.3, 9.3 (1)	9.85/9.85 ± 5.16, 6.2, 13.5 (2)
*P. nathusii*	Natural	—	3.0/2.77 ± 0.59, 2.1, 3.2 (3)
	Rural	7.9/8.01 ± 1.99, 4.8, 13.2 (27)	4.8/5.64 ± 2.39, 2.4, 13.4 (37)
*P. k. lepidus*	Natural	10.5/10.5, 10.5, 10.5 (1)	2.7/2.7, 2.7, 2.7 (1)
	Rural	8.3/8.3 ± 0.4, 7.9, 8.7 (3)	6.0/7.5 ± 4.44, 4.0, 12.5 (3)
	Urban	9.8/9.8, 9.8, 9.8 (1)	5.0/5.0, 5.0, 5.0 (1)
*Pl. auritus*	Natural	14.3/13.08 ± 3.41, 6.4, 16.0 (6)	2.6/2.81 ± 1.12, 1.7, 5.3 (9)
	Rural	12.6/11.64 ± 3.05, 6.3, 14.0 (5)	2.45/3.14 ± 3.3, 0.2, 10.4 (8)
*V. murinus*	Natural	—	10.5/10.5, 10.5, 10.5 (1)
	Rural	11.15/11.14 ± 2.3, 7.0, 14.8 (18)	6.7/6.24 ± 2.04, 2.7, 9.7 (23)

A complete description of all variables, including units and data types, is provided in the accompanying metadata file. All data are structured to facilitate comparative eco-immunological analyses across species, seasons, and ecological contexts.

## Technical Validation

Blood sampling and analysis followed standardized protocols to ensure data quality. Cell counts were performed manually using Goryaev chambers under consistent magnification. Smears were stained with Romanowsky-Giemsa and analyzed by a single trained observer to minimize variability.

The dataset^17^ distinguishes between missing and zero values: “NA” indicates the parameter was not evaluated, while “0” reflects the absence of the respective cell type. All numeric fields were validated for consistency and formatting.

This dataset reflects ostensibly healthy individuals because bats with signs of disease, trauma, severe dehydration, emaciation, or very young age were excluded by design. Thus, the reported distributions should not be interpreted as diagnostic reference intervals for sick animals. At the same time, we currently have no evidence that hemograms of clinically ill bats fall outside these ranges.

## Usage Notes

This dataset^17^ addresses that gap by providing large-scale data on blood parameters and leukocyte profiles for European bats, creating a resource for future research in eco-immunology, conservation, and disease ecology. Researchers should note that values summarize apparently healthy bats.

Researchers should consider the seasonal and urban context of sampling, as most data were collected during the hibernation season (Table 8, Supplementary Table S3). Manual smear analysis was conducted by a single observer, improving consistency but with typical limitations of visual classification. The main focus of this study was on qualitative and quantitative aspects of leukocytes, since they are effectors of both cellular and humoral links of the immune response. The dependence of the above-mentioned parameters on the species, age, and sex of bats was also traced (Tables 3, 4)^17^.

**Table 8:** Summary of mean and median values of RBC (red blood cells, 10^12^ cells/L) and WBC (white blood cells, 10^9^ cells/L) for different periods of Bat Life Seasons of seven bat species, with sample size more than 25 individuals (in total) (n = number of individuals).

Bat species name	Bat Life Seasons	RBC median/mean ± SD, min, max (n)	WBC median/mean ± SD, min, max (n)
*N. noctula*	Autumn Swarming	4.5/5.7 ± 3.17, 3.3, 9.3 (3)	5.0/4.6 ± 0.87, 3.6, 5.2 (3)
	Breeding	10.4/11.15 ± 3.03, 7.0, 23.0 (30)	4.9/5.94 ± 4.22, 1.4, 20.2 (54)
	Hibernation	10.5/9.9 ± 2.36, 6.0, 13.0 (20)	3.7/4.57 ± 3.22, 0.0, 13.9 (80)
*E. serotinus*	Autumn Swarming	6.0/6.79 ± 2.43, 4.5, 11.3 (9)	3.2/3.76 ± 1.79, 2.1, 7.6 (9)
	Breeding	8.85/8.44 ± 2.73, 3.6, 14.8 (38)	5.0/5.5 ± 2.92, 0.9, 12.0 (47)
	Hibernation	—	4.8/6.14 ± 2.63, 3.7, 11.0 (9)
*M. daubentonii*	Autumn Swarming	10.15/10.45 ± 1.97, 7.5, 13.3 (10)	3.7/4.33 ± 2.5, 0.8, 11.6 (20)
	Breeding	9.3/9.3, 9.3, 9.3 (1)	6.2/7.97 ± 4.9, 4.2, 13.5 (3)
*P. nathusii*	Breeding	7.9/8.01 ± 1.99, 4.8, 13.2 (27)	4.8/5.42 ± 2.42, 2.1, 13.4 (40)
*P. k. lepidus*	Autumn Swarming	10.5/10.5 ± nan, 10.5, 10.5 (1)	2.7/2.7 ± nan, 2.7, 2.7 (1)
	Breeding	8.5/8.68 ± 0.82, 7.9, 9.8 (4)	5.5/6.88 ± 3.84, 4.0, 12.5 (4)
*Pl. auritus*	Autumn Swarming	14.3/13.08 ± 3.41, 6.4, 16.0 (6)	2.6/2.81 ± 1.12, 1.7, 5.3 (9)
	Breeding	12.6/11.64 ± 3.05, 6.3, 14.0 (5)	2.45/3.14 ± 3.3, 0.2, 10.4 (8)
*V. murinus*	Autumn Swarming	—	10.5/10.5 ± nan, 10.5, 10.5 (1)
	Breeding	11.15/11.14 ± 2.3, 7.0, 14.8 (18)	6.7/6.24 ± 2.04, 2.7, 9.7 (23)

## Supplementary information

Supplementary tables

## Data Availability

The dataset described in this Data Descriptor is publicly available at Zenodo: 10.5281/zenodo.15648835. The dataset is provided in machine-readable formats with complete metadata and variable descriptions.

## Code availability

No custom code was used for data curation or validation. All analyses and summaries reported here can be reproduced directly from the deposited dataset using standard spreadsheets or statistical software.
