# Supplementary material for: Dataset on blood parameters including leukocyte profiles of eastern European vespertilionid and rhinolophid bats
Source: Sci Data. 2025 Dec 10;12:1994. doi: 10.1038/s41597-025-06274-0 (PMC12728163; doi:10.1038/s41597-025-06274-0)
Supplement: Supplementary file 1 — Supplementary tables [file 41597_2025_6274_MOESM1_ESM.pdf]

Supporting information for

Dataset on blood parameters including leukocyte profiles of eastern European vespertilionid and rhinolophid bats

Ihor Tovstukha [1,5], Valeriia Bohodist [1,2], Denys Muzyka [4], Viktoriia Radchuk [3], Kseniia Kravchenko [1,6], Anton Vlaschenko [1,2,3,4]*

[1] Ukrainian Bat Rehabilitation Center, NGO “Ukrainian Independent Ecology Institute”, Heorgia Tarasenko st., 40, 61001 Kharkiv, Ukraine
[2] Educational and Research Bat Biology Laboratory, H.S. Skovoroda Kharkiv National Pedagogical University, Institute of Natural Sciences, Valentynivska St. 2, Kharkiv, 61168, Ukraine
[3] Leibniz Institute for Zoo and Wildlife Research, Alfred-Kowalke-Straße 17, 10315, Berlin, Germany
[4] National Scientific Center “Institute of Experimental and Clinical Veterinary Medicine”, H. Skovorody St., 83, Kharkiv 61023, Ukraine
[5] Kharkiv International Medical University, Molochna St, 38, 61001, Kharkiv, Ukraine
[6] Faculty of Science, Technology and Medicine, University of Luxembourg, Luxembourg

*Correspondence: anton.vlaschenko@gmail.com

Contents of this file:

Table S1 Summary of mean and median values (%) of Leukocyte profiles (SN - segmented neutrophils; BN - band neutrophils; L - lymphocytes; M - monocytes; EO - eosinophils; BA - basophils) for different sex-age groups of seven bat species, with a sample size of more than 25 individuals (in total) (n=number of individuals)

Table S2 Summary of mean and median values (%) of Leukocyte profiles (SN - segmented neutrophils; BN - band neutrophils; L - lymphocytes; M - monocytes; EO - eosinophils; BA - basophils) for different Habitats of seven bat species, with a sample size of more than 25 individuals (in total) (n=number of individuals)

Table S3 Summary of mean and median values (%) of Leukocyte profiles (SN - segmented neutrophils; BN - band neutrophils; L - lymphocytes; M - monocytes; EO - eosinophils; BA - basophils) for different Bat Life Seasons of seven bat species, with a sample size of more than 25 individuals (in total) (n=number of individuals)

Supplementary tables

Supplementary Table S1 Summary of mean and median values (%) of Leukocyte profiles (SN - segmented neutrophils; BN - band neutrophils; L - lymphocytes; M - monocytes; EO - eosinophils; BA - basophils) for different sex-age groups of seven bat species, with a sample size of more than 25 individuals (in total) (n=number of individuals)

Bat species name	Sex and age group	SN% median/mean± SD, min, max	BN% median/mean± SD, min, max	L% median/mean± SD, min, max	M% median/mean± SD, min, max	EO% median/mean± SD, min, max	BA% median/mean± SD, min, max
N. noctula	F_total	37.5/40.4±18.0, 9.0, 87.0 (156)	1.0/1.2±1.1, 0.0, 8.0 (156)	59.0/54.9±18.5, 6.0, 90.0 (156)	2.0/3.0±2.4, 0.0, 12.0 (156)	0.0/0.5±0.6, 0.0, 2.0 (156)	0.0/0.3±0.5, 0.0, 2.0 (156)
	F_ad	46.0/43.7±19.2, 9.0, 87.0 (85)	1.0/1.1±0.9, 0.0, 5.0 (85)	52.0/52.2±19.7, 6.0, 90.0 (85)	2.0/2.6±2.2, 0.0, 12.0 (85)	0.0/0.5±0.6, 0.0, 2.0 (85)	0.0/0.2±0.4, 0.0, 1.0 (85)
	F_sad	35.0/36.8±15.9, 11.0, 73.0 (66)	1.0/1.3±1.3, 0.0, 8.0 (66)	61.5/57.7±16.6, 23.0, 86.0 (66)	3.0/3.5±2.7, 0.0, 12.0 (66)	0.0/0.5±0.5, 0.0, 1.0 (66)	0.0/0.3±0.5, 0.0, 2.0 (66)
	M_total	43.0/44.5±17.3, 11.0, 88.0 (140)	1.0/1.2±0.9, 0.0, 4.0 (140)	52.0/50.6±18.3, 8.0, 86.0 (140)	3.0/3.2±2.3, 0.0, 12.0 (140)	0.0/0.4±0.8, 0.0, 6.0 (140)	0.0/0.2±0.5, 0.0, 2.0 (140)
	M_ad	46.0/47.3±17.6, 13.0, 88.0 (63)	1.0/1.0±0.8, 0.0, 4.0 (63)	50.0/47.7±19.2, 8.0, 85.0 (63)	3.0/3.4±2.6, 0.0, 12.0 (63)	0.0/0.5±0.9, 0.0, 6.0 (63)	0.0/0.2±0.5, 0.0, 2.0 (63)
	M_sad	40.5/42.3±17.0, 11.0, 83.0 (74)	1.0/1.3±1.0, 0.0, 4.0 (74)	53.5/52.9±17.6, 12.0, 86.0 (74)	3.0/3.0±2.0, 0.0, 11.0 (74)	0.0/0.4±0.6, 0.0, 2.0 (74)	0.0/0.3±0.5, 0.0, 2.0 (74)
E. serotinus	F_total	42.0/43.1±18.4, 6.0, 74.0 (45)	1.0/1.2±1.1, 0.0, 5.0 (45)	54.0/52.3±18.3, 22.0, 93.0 (45)	2.0/2.8±2.9, 0.0, 13.0 (45)	1.0/0.6±0.6, 0.0, 2.0 (45)	0.0/0.1±0.3, 0.0, 1.0 (45)
	F_ad	42.0/44.1±17.7, 12.0, 74.0 (36)	1.0/1.1±1.0, 0.0, 4.0 (36)	53.0/51.9±18.0, 22.0, 84.0 (36)	2.0/2.1±1.9, 0.0, 10.0 (36)	1.0/0.7±0.6, 0.0, 2.0 (36)	0.0/0.1±0.3, 0.0, 1.0 (36)
	F_sad	36.0/38.9±21.7, 6.0, 74.0 (9)	1.0/1.6±1.5, 0.0, 5.0 (9)	54.0/53.6±20.3, 25.0, 93.0 (9)	3.0/5.4±4.4, 1.0, 13.0 (9)	0.0/0.4±0.5, 0.0, 1.0 (9)	0.0/0.1±0.3, 0.0, 1.0 (9)
	M_total	40.0/38.9±16.6, 12.0, 80.0 (59)	1.0/1.5±1.3, 0.0, 5.0 (59)	53.0/53.8±17.6, 18.0, 81.0 (59)	4.0/5.3±3.4, 1.0, 15.0 (59)	0.0/0.5±0.8, 0.0, 4.0 (59)	0.0/0.2±0.4, 0.0, 1.0 (59)
	M_ad	41.0/38.3±15.6, 12.0, 75.0 (49)	1.0/1.7±1.3, 0.0, 5.0 (49)	50.0/53.7±17.4, 20.0, 81.0 (49)	5.0/5.7±3.5, 1.0, 15.0 (49)	0.0/0.5±0.8, 0.0, 4.0 (49)	0.0/0.2±0.4, 0.0, 1.0 (49)
	M_sad	36.0/37.6±17.5, 15.0, 73.0 (9)	1.0/0.9±0.8, 0.0, 2.0 (9)	59.0/58.6±15.9, 34.0, 80.0 (9)	3.0/3.7±1.9, 2.0, 8.0 (9)	0.0/0.4±0.5, 0.0, 1.0 (9)	0.0/0.1±0.3, 0.0, 1.0 (9)

Table S1 (continued)

Bat species name	Sex and age group	SN% median/mean± SD, min, max	BN% median/mean± SD, min, max	L% median/mean± SD, min, max	M% median/mean± SD, min, max	EO% median/mean± SD, min, max	BA% median/mean± SD, min, max
M. daubentonii	F_total	34.0/37.0±16.1, 15.0, 70.0 (21)	1.0/0.7±0.7, 0.0, 2.0 (21)	63.0/57.8±17.0, 24.0, 79.0 (21)	2.0/3.8±3.0, 1.0, 11.0 (21)	0.0/0.5±0.7, 0.0, 2.0 (21)	0.0/0.2±0.4, 0.0, 1.0 (21)
	F_ad	24.5/30.6±12.1, 15.0, 50.0 (10)	0.0/0.4±0.5, 0.0, 1.0 (10)	72.5/65.8±12.3, 43.0, 77.0 (10)	2.0/2.2±1.3, 1.0, 5.0 (10)	0.0/0.6±0.8, 0.0, 2.0 (10)	0.0/0.4±0.5, 0.0, 1.0 (10)
	F_sad	45.0/44.3±17.8, 20.0, 70.0 (10)	1.0/1.0±0.8, 0.0, 2.0 (10)	48.5/48.7±17.7, 24.0, 79.0 (10)	5.5/5.6±3.5, 1.0, 11.0 (10)	0.0/0.3±0.5, 0.0, 1.0 (10)	0.0/0.1±0.3, 0.0, 1.0 (10)
	M_total	35.0/36.8±14.4, 10.0, 69.0 (59)	1.0/0.9±0.8, 0.0, 4.0 (59)	62.0/59.0±15.2, 23.0, 85.0 (59)	2.0/2.6±2.1, 0.0, 8.0 (59)	0.0/0.4±0.7, 0.0, 3.0 (59)	0.0/0.2±0.4, 0.0, 1.0 (59)
	M_ad	34.5/36.8±13.9, 10.0, 69.0 (36)	1.0/0.9±0.7, 0.0, 3.0 (36)	60.0/58.9±14.7, 23.0, 85.0 (36)	2.0/2.5±2.1, 0.0, 8.0 (36)	1.0/0.6±0.7, 0.0, 3.0 (36)	0.0/0.2±0.4, 0.0, 1.0 (36)
	M_sad	35.0/37.6±15.9, 13.0, 63.0 (21)	1.0/1.1±0.9, 0.0, 4.0 (21)	58.0/58.1±16.4, 34.0, 85.0 (21)	2.0/2.7±2.2, 0.0, 8.0 (21)	0.0/0.2±0.5, 0.0, 2.0 (21)	0.0/0.2±0.4, 0.0, 1.0 (21)
P. nathusii	F_total	32.5/34.9±12.6, 16.0, 64.0 (24)	2.0/1.8±1.1, 0.0, 4.0 (24)	63.0/58.2±13.8, 27.0, 82.0 (24)	4.0/4.2±2.2, 1.0, 10.0 (24)	0.0/0.3±0.6, 0.0, 2.0 (24)	0.0/0.5±0.7, 0.0, 2.0 (24)
	F_ad	41.5/40.7±16.5, 16.0, 64.0 (6)	1.5/1.7±1.2, 0.0, 3.0 (6)	52.0/53.8±18.9, 27.0, 82.0 (6)	2.5/2.8±1.5, 1.0, 5.0 (6)	0.0/0.3±0.5, 0.0, 1.0 (6)	0.5/0.7±0.8, 0.0, 2.0 (6)
	F_sad	31.0/33.0±10.9, 18.0, 63.0 (18)	2.0/1.8±1.0, 0.0, 4.0 (18)	63.0/59.7±12.0, 29.0, 77.0 (18)	4.0/4.7±2.3, 2.0, 10.0 (18)	0.0/0.3±0.7, 0.0, 2.0 (18)	0.0/0.5±0.6, 0.0, 2.0 (18)
	M_total	36.0/35.4±11.6, 16.0, 53.0 (34)	1.0/1.1±1.2, 0.0, 5.0 (34)	59.0/60.1±11.8, 39.0, 82.0 (34)	2.0/2.6±2.1, 0.0, 9.0 (34)	0.0/0.3±0.5, 0.0, 2.0 (34)	0.0/0.3±0.5, 0.0, 2.0 (34)
	M_ad	43.5/38.7±11.9, 21.0, 53.0 (20)	1.0/0.8±0.8, 0.0, 3.0 (20)	54.0/58.3±12.0, 43.0, 79.0 (20)	1.0/1.6±1.5, 0.0, 6.0 (20)	0.0/0.2±0.4, 0.0, 1.0 (20)	0.0/0.2±0.6, 0.0, 2.0 (20)
	M_sad	28.5/28.8±9.0, 16.0, 47.0 (12)	1.0/1.8±1.5, 0.0, 5.0 (12)	64.0/64.2±11.5, 39.0, 82.0 (12)	4.5/4.5±2.0, 2.0, 9.0 (12)	0.0/0.3±0.5, 0.0, 1.0 (12)	0.5/0.5±0.5, 0.0, 1.0 (12)

Table S1 (continued)

Bat species name	Sex and age group	SN% median/mean± SD, min, max	BN% median/mean± SD, min, max	L% median/mean± SD, min, max	M% median/mean± SD, min, max	EO% median/mean± SD, min, max	BA% median/mean± SD, min, max
P. k. lepidus	F_total	66.5/64.7±13.2, 33.0, 89.0 (38)	1.0/0.8±0.6, 0.0, 2.0 (38)	30.0/31.3±13.6, 7.0, 64.0 (38)	2.0/2.4±1.7, 1.0, 7.0 (38)	0.0/0.3±0.5, 0.0, 1.0 (38)	0.0/0.4±0.5, 0.0, 2.0 (38)
	F_ad	68.5/66.5±13.1, 33.0, 89.0 (26)	1.0/0.8±0.5, 0.0, 2.0 (26)	28.0/29.7±13.5, 7.0, 64.0 (26)	2.0/2.4±1.6, 1.0, 7.0 (26)	0.0/0.2±0.4, 0.0, 1.0 (26)	0.0/0.3±0.5, 0.0, 1.0 (26)
	F_sad	58.5/60.8±13.2, 41.0, 79.0 (12)	1.0/0.8±0.7, 0.0, 2.0 (12)	37.0/34.8±14.0, 18.0, 57.0 (12)	2.0/2.6±2.0, 1.0, 7.0 (12)	0.5/0.5±0.5, 0.0, 1.0 (12)	0.0/0.5±0.7, 0.0, 2.0 (12)
	M_total	57.5/56.8±16.8, 29.0, 88.0 (12)	1.0/0.8±0.8, 0.0, 2.0 (12)	37.5/38.3±17.2, 9.0, 66.0 (12)	2.0/3.2±2.2, 1.0, 7.0 (12)	0.0/0.5±0.8, 0.0, 2.0 (12)	0.0/0.3±0.5, 0.0, 1.0 (12)
	M_ad	61.0/63.1±14.4, 44.0, 88.0 (7)	1.0/1.0±0.8, 0.0, 2.0 (7)	31.0/30.3±13.1, 9.0, 47.0 (7)	5.0/4.3±2.2, 2.0, 7.0 (7)	1.0/0.9±0.9, 0.0, 2.0 (7)	0.0/0.4±0.5, 0.0, 1.0 (7)
	M_sad	40.5/41.8±11.9, 29.0, 57.0 (4)	0.5/0.5±0.6, 0.0, 1.0 (4)	58.5/55.5±11.9, 39.0, 66.0 (4)	1.5/2.0±1.4, 1.0, 4.0 (4)	0.0/0.0±0.0, 0.0, 0.0 (4)	0.0/0.2±0.5, 0.0, 1.0 (4)
Pl. auritus	F_total	40.0/41.3±13.4, 22.0, 68.0 (15)	1.0/1.1±1.1, 0.0, 3.0 (15)	55.0/53.4±14.0, 27.0, 72.0 (15)	2.0/3.6±3.1, 1.0, 11.0 (15)	0.0/0.2±0.4, 0.0, 1.0 (15)	0.0/0.3±0.5, 0.0, 1.0 (15)
	F_ad	38.0/39.3±12.5, 23.0, 59.0 (9)	1.0/0.9±1.1, 0.0, 3.0 (9)	57.0/56.0±12.7, 36.0, 72.0 (9)	2.0/3.3±3.1, 1.0, 11.0 (9)	0.0/0.1±0.3, 0.0, 1.0 (9)	0.0/0.2±0.4, 0.0, 1.0 (9)
	F_sad	42.0/43.0±16.8, 22.0, 68.0 (5)	2.0/1.4±1.3, 0.0, 3.0 (5)	55.0/51.8±17.0, 27.0, 71.0 (5)	2.0/2.8±1.6, 1.0, 5.0 (5)	0.0/0.4±0.5, 0.0, 1.0 (5)	1.0/0.6±0.5, 0.0, 1.0 (5)
	M_total	28.0/26.5±11.6, 7.0, 52.0 (21)	1.0/1.0±1.1, 0.0, 3.0 (21)	66.0/68.7±13.3, 45.0, 91.0 (21)	2.0/3.5±2.8, 1.0, 10.0 (21)	0.0/0.3±0.6, 0.0, 2.0 (21)	0.0/0.0±0.2, 0.0, 1.0 (21)
	M_ad	30.5/27.5±13.3, 8.0, 52.0 (12)	0.0/0.6±0.9, 0.0, 2.0 (12)	65.0/67.8±15.1, 45.0, 90.0 (12)	2.5/4.0±3.2, 1.0, 10.0 (12)	0.0/0.2±0.4, 0.0, 1.0 (12)	0.0/0.0±0.0, 0.0, 0.0 (12)
	M_sad	26.0/24.7±10.3, 7.0, 38.0 (6)	1.5/1.3±0.8, 0.0, 2.0 (6)	69.0/70.3±12.2, 57.0, 91.0 (6)	2.5/3.3±2.3, 1.0, 7.0 (6)	0.0/0.3±0.5, 0.0, 1.0 (6)	0.0/0.0±0.0, 0.0, 0.0 (6)

Table S1 (continued)

Bat species name	Sex and age group	SN% median/mean± SD, min, max	BN% median/mean± SD, min, max	L% median/mean± SD, min, max	M% median/mean± SD, min, max	EO% median/mean± SD, min, max	BA% median/mean± SD, min, max
V. murinus	F_total	27.0/30.7±16.6, 9.0, 63.0 (15)	1.0/1.5±1.1, 0.0, 4.0 (15)	67.0/62.7±16.6, 34.0, 82.0 (15)	4.0/4.5±2.2, 2.0, 9.0 (15)	0.0/0.3±0.6, 0.0, 2.0 (15)	0.0/0.3±0.5, 0.0, 1.0 (15)
	F_ad	39.0/39.0±17.0, 27.0, 51.0 (2)	0.5/0.5±0.7, 0.0, 1.0 (2)	54.5/54.5±20.5, 40.0, 69.0 (2)	5.5/5.5±3.5, 3.0, 8.0 (2)	0.5/0.5±0.7, 0.0, 1.0 (2)	0.0/0.0±0.0, 0.0, 0.0 (2)
	F_sad	28.0/30.0±18.4, 9.0, 63.0 (11)	2.0/1.7±1.2, 0.0, 4.0 (11)	67.0/63.3±18.0, 34.0, 82.0 (11)	4.0/4.4±2.1, 2.0, 9.0 (11)	0.0/0.2±0.6, 0.0, 2.0 (11)	0.0/0.5±0.5, 0.0, 1.0 (11)
	M_total	27.0/29.7±10.4, 15.0, 46.0 (13)	2.0/2.4±1.6, 0.0, 5.0 (13)	66.0/62.8±14.3, 36.0, 80.0 (13)	3.0/3.7±1.7, 1.0, 7.0 (13)	0.0/0.4±0.7, 0.0, 2.0 (13)	0.0/0.3±0.6, 0.0, 2.0 (13)
	M_ad	28.0/30.3±8.7, 23.0, 40.0 (3)	3.0/2.7±1.5, 1.0, 4.0 (3)	66.0/58.7±20.0, 36.0, 74.0 (3)	3.0/3.7±2.1, 2.0, 6.0 (3)	0.0/0.7±1.2, 0.0, 2.0 (3)	0.0/0.7±1.2, 0.0, 2.0 (3)
	M_sad	31.0/32.9±9.9, 21.0, 46.0 (8)	2.0/2.5±1.8, 0.0, 5.0 (8)	60.0/60.4±12.2, 44.0, 77.0 (8)	3.5/3.9±1.8, 1.0, 7.0 (8)	0.0/0.2±0.5, 0.0, 1.0 (8)	0.0/0.1±0.4, 0.0, 1.0 (8)

Table S2 Summary of mean and median values (%) of Leukocyte profiles (SN - segmented neutrophils; BN - band neutrophils; L - lymphocytes; M - monocytes; EO - eosinophils; BA - basophils) for different Habitats of seven bat species, with a sample size of more than 25 individuals (in total) (n=number of individuals)

Bat species name	Habitat type	SN% median/mean±SD, min, max	BN% median/mean±SD, min, max	L% median/mean±SD, min, max	M% median/mean±SD, min, max	EO% median/mean±SD, min, max	BA% median/mean±SD, min, max
N. noctula	Natural	47.0/46.93± 18.52, 9.0, 88.0 (74)	1.0/0.88±0.91, 0.0, 5.0 (74)	50.0/49.97 ±19.21, 8.0, 90.0 (74)	1.0/2.07±1.95, 0.0, 12.0 (74)	0.0/0.42 ±0.55, 0.0, 2.0 (74)	0.0/0.14±0.34, 0.0, 1.0 (74)
	Rural	48.0/47.26± 9.19, 29.0, 73.0 (23)	2.0/2.22± 1.28, 0.0, 5.0 (23)	42.0/43.04 ± 8.33, 23.0, 62.0 (23)	6.0/6.65± 2.81, 3.0, 12.0 (23)	0.0/0.39 ±0.66, 0.0, 2.0 (23)	0.0/0.43± 0.59, 0.0, 2.0 (23)
	Urban	36.0/39.6 ± 17.6, 10.0, 83.0 (193)	1.0/1.1 ± 0.8, 0.0, 4.0 (193)	60.0/55.55 ± 18.38, 10.0, 86.0 (193)	3.0/3.04 ± 2.02, 0.0, 12.0 (193)	0.0/0.49 ± 0.72, 0.0, 6.0 (193)	0.0/0.29 ± 0.48, 0.0, 2.0 (193)
E. serotinus	Natural	36.0/36.17 ±12.47, 15.0, 67.0 (36)	1.0/1.31±1.19, 0.0, 5.0 (36)	57.5/58.0±13.6, 30.0, 84.0 (36)	2.0/3.69±3.56, 0.0, 13.0 (36)	1.0/0.72 ±0.81, 0.0, 4.0 (36)	0.0/0.11± 0.32, 0.0, 1.0 (36)
	Rural	26.5/32.46 ±15.2, 12.0, 58.0 (24)	2.0/1.83±1.34, 0.0, 5.0 (24)	66.5/59.5±18.13, 28.0, 81.0 (24)	5.0/5.58±2.95, 2.0, 11.0 (24)	0.0/0.29 ±0.55, 0.0, 2.0 (24)	0.0/0.33±0.48, 0.0, 1.0 (24)
	Urban	47.5/48.95 ± 18.79, 6.0, 80.0 (44)	1.0/1.18 ± 1.15, 0.0, 4.0 (44)	41.5/45.68 ± 18.3, 18.0, 93.0 (44)	3.0/3.86 ± 3.31, 0.0, 15.0 (44)	0.0/0.52 ± 0.66, 0.0, 3.0 (44)	0.0/0.05 ± 0.21, 0.0, 1.0 (44)
M. daubentonii	Natural	34.5/36.87 ± 14.89, 10.0, 70.0 (78)	1.0/0.87 ± 0.78, 0.0, 4.0 (78)	62.0/58.67 ± 15.67, 23.0, 85.0 (78)	2.0/2.86 ± 2.46, 0.0, 11.0 (78)	0.0/0.46 ± 0.66, 0.0, 3.0 (78)	0.0/0.24 ± 0.43, 0.0, 1.0 (78)
	Rural	35.5/35.5 ± 13.44, 26.0, 45.0 (2)	1.5/1.5 ± 0.71, 1.0, 2.0 (2)	59.0/59.0 ± 14.14, 49.0, 69.0 (2)	4.0/4.0 ± 0.0, 4.0, 4.0 (2)	0.0/0.0 ± 0.0, 0.0, 0.0 (2)	0.0/0.0 ± 0.0, 0.0, 0.0 (2)
P. nathusii	Natural	42.0/41.11 ± 12.67, 21.0, 64.0 (19)	1.0/1.32 ± 1.25, 0.0, 4.0 (19)	52.0/54.68 ± 15.14, 27.0, 79.0 (19)	1.0/2.32 ± 2.85, 0.0, 10.0 (19)	0.0/0.47 ± 0.61, 0.0, 2.0 (19)	0.0/0.11 ± 0.32, 0.0, 1.0 (19)
	Rural	32.0/32.36 ± 10.56, 16.0, 53.0 (39)	1.0/1.46 ± 1.12, 0.0, 5.0 (39)	63.0/61.62 ± 10.58, 45.0, 82.0 (39)	4.0/3.77 ± 1.83, 1.0, 9.0 (39)	0.0/0.23 ± 0.54, 0.0, 2.0 (39)	0.0/0.56 ± 0.64, 0.0, 2.0 (39)

Table S2 (continued)

Bat species name	Habitat type	SN% median/mean±SD, min, max	BN% median/mean±SD, min, max	L% median/mean±SD, min, max	M% median/mean±SD, min, max	EO% median/mean±SD, min, max	BA% median/mean±SD, min, max
P. k. lepidus	Natural	61.0/61.0 ± nan, 61.0, 61.0 (1)	1.0/1.0 ± nan, 1.0, 1.0 (1)	31.0/31.0 ± nan, 31.0, 31.0 (1)	7.0/7.0 ± nan, 7.0, 7.0 (1)	0.0/0.0 ± nan, 0.0, 0.0 (1)	0.0/0.0 ± nan, 0.0, 0.0 (1)
	Rural	60.0/58.67 ± 4.16, 54.0, 62.0 (3)	1.0/1.33 ± 0.58, 1.0, 2.0 (3)	31.0/33.67 ± 7.37, 28.0, 42.0 (3)	6.0/5.0 ± 2.65, 2.0, 7.0 (3)	1.0/0.67 ± 0.58, 0.0, 1.0 (3)	1.0/0.67 ± 0.58, 0.0, 1.0 (3)
	Urban	66.0/63.56 ±14.83, 29.0, 89.0 (45)	1.0/0.76± 0.61, 0.0, 2.0 (45)	30.0/32.67 ±15.28, 7.0, 66.0 (45)	2.0/2.31± 1.52, 1.0, 7.0 (45)	0.0/0.31 ±0.51, 0.0, 2.0 (45)	0.0/0.38± 0.53, 0.0, 2.0 (45)
Pl. auritus	Natural	33.5/31.5± 14.07, 7.0, 59.0 (24)	1.0/0.79 ± 0.83, 0.0, 2.0 (24)	62.0/63.62 ±15.84, 36.0, 91.0 (24)	2.0/3.71± 3.37, 1.0, 11.0 (24)	0.0/0.29 ±0.55, 0.0, 2.0 (24)	0.0/0.08± 0.28, 0.0, 1.0 (24)
	Rural	31.5/34.92 ±14.89, 13.0, 68.0 (12)	2.0/1.67± 1.3, 0.0, 3.0 (12)	61.0/59.67 ±14.87, 27.0, 83.0 (12)	4.0/3.25± 1.42, 1.0, 5.0 (12)	0.0/0.17 ±0.39, 0.0, 1.0 (12)	0.0/0.33± 0.49, 0.0, 1.0 (12)
V. murinus	Natural	43.0/43.0± nan, 43.0, 43.0 (1)	2.0/2.0± nan, 2.0, 2.0 (1)	49.0/49.0± nan, 49.0, 49.0 (1)	5.0/5.0± nan, 5.0, 5.0 (1)	1.0/1.0± nan, 1.0, 1.0 (1)	0.0/0.0± nan, 0.0, 0.0 (1)
	Rural	27.0/27.83 ±11.96, 9.0, 56.0 (24)	2.0/2.12± 1.33, 0.0, 5.0 (24)	66.5/64.88 ±13.9, 34.0, 82.0 (24)	4.0/4.12± 1.8, 2.0, 9.0 (24)	0.0/0.29 ±0.62, 0.0, 2.0 (24)	0.0/0.33± 0.56, 0.0, 2.0 (24)
	Urban	42.5/42.5± 28.99, 22.0, 63.0 (2)	0.0/0.0± 0.0, 0.0, 0.0 (2)	55.5/55.5± 30.41, 34.0, 77.0 (2)	1.5/1.5± 0.71, 1.0, 2.0 (2)	0.0/0.0± 0.0, 0.0, 0.0 (2)	0.5/0.5± 0.71, 0.0, 1.0 (2)

Table S3 Summary of mean and median values (%) of Leukocyte profiles (SN - segmented neutrophils; BN - band neutrophils; L - lymphocytes; M - monocytes; EO - eosinophils; BA - basophils) for different Bat Life Seasons of seven bat species, with a sample size of more than 25 individuals (in total) (n=number of individuals)

Bat species name	Bat Life Seasons	SN% median/mean±SD, min, max	BN% median/mean±SD, min, max	L% median/mean±SD, min, max	M% median/mean±SD, min, max	EO% median/mean±SD, min, max	BA% median/mean±SD, min, max
N. noctula	Autumn Swarming	54.5/56.0 ±8.04, 49.0, 66.0 (4)	1.0/1.5± 1.0, 1.0, 3.0 (4)	37.5/37.5± 9.68, 27.0, 48.0 (4)	4.5/4.25± 2.75, 1.0, 7.0 (4)	0.5/0.5± 0.58, 0.0, 1.0 (4)	0.0/0.25± 0.5, 0.0, 1.0 (4)
	Breeding	48.0/47.43±16.6, 9.0, 88.0 (101)	1.0/1.21± 1.16, 0.0, 5.0 (101)	44.0/47.85 ±17.29, 8.0, 90.0 (101)	2.0/3.23± 2.89, 0.0, 12.0 (101)	0.0/0.38 ±0.56, 0.0, 2.0 (101)	0.0/0.21± 0.43, 0.0, 2.0 (101)
	Hibernation	35.0/38.86 ± 17.54, 10.0, 83.0 (185)	1.0/1.09 ± 0.78, 0.0, 4.0 (185)	61.0/56.36 ±18.3, 10.0, 86.0 (185)	3.0/2.97± 2.01, 0.0, 12.0 (185)	0.0/0.51 ±0.72, 0.0, 6.0 (185)	0.0/0.29± 0.48, 0.0, 2.0 (185)
E. serotinus	Autumn Swarming	40.0/38.15 ±9.51, 18.0, 51.0 (13)	2.0/2.08± 1.38, 1.0, 5.0 (13)	53.0/52.31 ±8.55, 40.0, 69.0 (13)	8.0/6.69± 4.13, 0.0, 13.0 (13)	1.0/0.62 ±0.65, 0.0, 2.0 (13)	0.0/0.15± 0.38, 0.0, 1.0 (13)
	Breeding	38.0/37.26 ±15.78, 12.0, 75.0 (62)	1.0/1.55± 1.24, 0.0, 5.0 (62)	56.5/55.95 ±17.38, 20.0, 84.0 (62)	3.0/4.58± 3.48, 0.0, 15.0 (62)	0.0/0.5 0.78, 0.0, 4.0 (62)	0.0/0.16 ± 0.37, 0.0, 1.0 (62)
	Hibernation	54.0/49.28 ± 20.78, 6.0, 80.0 (29)	1.0/0.69 ± 0.76, 0.0, 3.0 (29)	41.0/47.48 ± 20.69, 18.0, 93.0 (29)	2.0/2.28 ± 1.1, 1.0, 4.0 (29)	1.0/0.59 ± 0.57, 0.0, 2.0 (29)	0.0/0.07 ± 0.26, 0.0, 1.0 (29)
M. daubentonii	Autumn Swarming	33.0/36.16 ± 15.87, 10.0, 70.0 (58)	1.0/0.84 ± 0.72, 0.0, 3.0 (58)	62.5/58.93 ± 16.73, 23.0, 85.0 (58)	3.0/3.4 ± 2.56, 0.0, 11.0 (58)	0.0/0.45 ± 0.71, 0.0, 3.0 (58)	0.0/0.22 ± 0.42, 0.0, 1.0 (58)
	Breeding	40.5/38.64 ± 11.59, 20.0, 63.0 (22)	1.0/1.0 ± 0.93, 0.0, 4.0 (22)	56.5/58.0 ± 12.23, 34.0, 79.0 (22)	1.5/1.55 ± 1.41, 0.0, 4.0 (22)	0.0/0.45 ± 0.51, 0.0, 1.0 (22)	0.0/0.27 ± 0.46, 0.0, 1.0 (22)

Table S3 (continued)

Bat species name	Bat Life Seasons	SN% median/mean±SD, min, max	BN% median/mean±SD, min, max	L% median/mean±SD, min, max	M% median/mean±SD, min, max	EO% median/mean±SD, min, max	BA% median/mean±SD, min, max
P. nathusii	Breeding	33.5/35.22 ± 11.93, 16.0, 64.0 (58)	1.0/1.41 ± 1.16, 0.0, 5.0 (58)	61.5/59.34 ± 12.56, 27.0, 82.0 (58)	3.0/3.29 ± 2.29, 0.0, 10.0 (58)	0.0/0.31 ± 0.57, 0.0, 2.0 (58)	0.0/0.41 ± 0.59, 0.0, 2.0 (58)
P. k. lepidus	Autumn Swarming	61.0 (1)	1.0 (1)	31.0 (1)	7.0 (1)	0.0 (1)	0.0 (1)
	Breeding	59.0/58.5 ± 3.42, 54.0, 62.0 (4)	1.0/1.0 ± 0.82, 0.0, 2.0 (4)	33.5/34.25 ± 6.13, 28.0, 42.0 (4)	5.5/5.0 ± 2.16, 2.0, 7.0 (4)	0.5/0.5 ± 0.58, 0.0, 1.0 (4)	1.0/0.75 ± 0.5, 0.0, 1.0 (4)
	Hibernation	66.5/63.68 ± 14.97, 29.0, 89.0 (44)	1.0/0.77 ± 0.6, 0.0, 2.0 (44)	30.0/32.59 ± 15.45, 7.0, 66.0 (44)	2.0/2.25 ± 1.48, 1.0, 7.0 (44)	0.0/0.32 ± 0.52, 0.0, 2.0 (44)	0.0/0.36 ± 0.53, 0.0, 2.0 (44)
Pl. auritus	Autumn Swarming	33.5/31.64 ± 14.39, 7.0, 59.0 (22)	1.0/0.86 ± 0.83, 0.0, 2.0 (22)	62.0/63.32 ± 16.25, 36.0, 91.0 (22)	2.0/3.82 ± 3.5, 1.0, 11.0 (22)	0.0/0.27 ± 0.55, 0.0, 2.0 (22)	0.0/0.09 ± 0.29, 0.0, 1.0 (22)
	Breeding	31.5/34.21 ± 14.36, 13.0, 68.0 (14)	2.0/1.43 ± 1.34, 0.0, 3.0 (14)	61.0/60.71 ± 14.48, 27.0, 83.0 (14)	3.5/3.14 ± 1.35, 1.0, 5.0 (14)	0.0/0.21 ± 0.43, 0.0, 1.0 (14)	0.0/0.29 ± 0.47, 0.0, 1.0 (14)
V. murinus	Autumn Swarming	43.0 (1)	2.0 (1)	49.0 (1)	5.0 (1)	1.0 (1)	0.0 (1)
	Breeding	27.0/27.83 ± 11.96, 9.0, 56.0 (24)	2.0/2.12 ± 1.33, 0.0, 5.0 (24)	66.5/64.88 ± 13.9, 34.0, 82.0 (24)	4.0/4.12 ± 1.8, 2.0, 9.0 (24)	0.0/0.29 ± 0.62, 0.0, 2.0 (24)	0.0/0.33 ± 0.56, 0.0, 2.0 (24)
	Hibernation	42.5/42.5 ± 28.99, 22.0, 63.0 (2)	0.0/0.0 ± 0.0, 0.0, 0.0 (2)	55.5/55.5 ± 30.41, 34.0, 77.0 (2)	1.5/1.5 ± 0.71, 1.0, 2.0 (2)	0.0/0.0 ± 0.0, 0.0, 0.0 (2)	0.5/0.5 ± 0.71, 0.0, 1.0 (2)
